# Wangbi granule as a combination therapy to achieve clinical deep remission in rheumatoid arthritis: protocol for a multicenter, triple-blind, randomised, placebo-controlled trial

**DOI:** 10.1186/s13020-023-00728-6

**Published:** 2023-02-28

**Authors:** Jinping Wang, Zihan Wang, Tianyi Lan, Liubo Zhang, Zhenbin Li, Xinchang Wang, Qinghua Zou, Yuan Wang, Yanqi Li, Ruili Luo, Nan Zhang, Yuan Xu, Mengtao Li, Qingwen Tao

**Affiliations:** 1 Traditional Chinese Medicine Department of Rheumatology, China-Japan Friendship Hospital, Beijing, People’s Republic of China; 2 Beijing University of Chinese Medicine, Beijing, People’s Republic of China; 3 Department of Rheumatology and Immunology, The 980th Hospital of PLA Joint Logistics Support Force, Shijiazhuang, People’s Republic of China; 4 Department of Rheumatology and Immunology, The Second Affiliated Hospital of Zhejiang Chinese Medical University, Hangzhou, People’s Republic of China; 5 Department of Traditional Chinese Medicine and Rheumatology, The First Hospital Affiliated to Army Medical University, Chongqing, People’s Republic of China; 6 Department of Rheumatology, The First Affiliated Hospital of Anhui University of Traditional Chinese Medicine, Hefei, People’s Republic of China; 7 Department of Rheumatology and Immunology, Peking Union Medical College Hospital, Beijing, People’s Republic of China

**Keywords:** Rheumatoid arthritis, Wangbi granule, Clinical deep remission, Efficacy, Randomized controlled trial, Protocol

## Abstract

**Introduction:**

Rheumatoid arthritis (RA) is a chronic inflammatory autoimmune disease that may lead to bone erosion and disability. Although there are many biological therapies in RA treatment nowadays, such as etanercept and tofacitinib, there are still a considerable number of patients who cannot achieve clinical deep remission, which makes patients feel pain and stiffness of joints. As a traditional Chinese medicine preparation, Wangbi granule showed a synergistic role with methotrexate in the treatment of RA patients with “kidney deficiency and dampness” or “stasis blocking channels”. Therefore, it is a promising therapeutic strategy for the clinical deep remission of RA. In this study, Wangbi granule will be used as the test drug. The investigators conduct this study to evaluate the efficacy and safety of Wangbi granule in the treatment of patients who have not achieved deep remission despite the use of methotrexate and tofacitinib.

**Methods and analysis:**

Two parallel randomized, triple-blind, placebo-controlled trials will be conducted. In six study centers, 340 eligible RA patients will be recruited and randomly allocated to either the intervention group or the control group (in a 1:1 ratio). They will receive Wangbi granule or Wangbi placebo 12.0 g each time, three times a day for 12 weeks. The primary outcome is the disease activity score derivative for 28 joints (DAS28). Secondary outcomes are patient-reported outcomes, American College of Rheumatology 50% response criteria (ACR50), fatigue scale-14 (FS-14), visual analogue scale for pain (VAS), health assessment questionnaire disability index (HAQ-DI) and biomarkers such as C-reactive protein (CRP) and erythrocyte sedimentation rate (ESR).

**Expected outcomes:**

The success of this study will provide strong evidence to confirm the efficacy and safety of Wangbi granule in the treatment of RA.

*Trial registration* The trial has been registered in the ClinicalTrials Registry (NCT05540938, Date: 09/15/2022, https://clinicaltrials.gov/ct2/show/NCT05540938)

## Background

Rheumatoid arthritis (RA) is a chronic inflammatory autoimmune disease and is currently one of the major disabling diseases worldwide, with a prevalence of about 1% [1, 2]. RA is mainly manifested as chronic synovial inflammation, which undergoes synovial proliferation, infiltration of immune and inflammatory cells, and eventually progresses to destruction of cartilage and bone, resulting in limited joint movement and physical disability [3]. Disease activity and bone erosion in RA have been the most important clinical concerns. Reducing disease activity, controlling synovial inflammation, and delaying bone destruction are the crucial issues that need to be addressed.

The standard of care for RA has gone through phases of hormones, non-steroidal antiinflammatory drugs (NSAIDs), conventional synthetic disease-modifying antirheumatic drugs (csDMARDs), biologic disease-modifying antirheumatic drugs (bDMARDs) and target-mediated drug for small molecules [4]. The prognosis of RA has been improved substantially due to the appearance of bDMARDs and target-mediated drug for small molecules. However, once RA is controlled, dose reduction or withdrawal of these drugs must be taken into consideration because of infection risk, dose-dependent adverse events, or treatment cost. More importantly, during the treatment process, even after intensive Western medication, there are a considerable number of patients who cannot achieve clinical deep remission, which makes patients still feel pain and stiffness of joints [5], and gradually lead to a series of complications such as chronic fatigue and diffuse pain [6, 7]. In the case of the methotrexate combined with tofacitinib regimen, for example, a study demonstrated that even with the application of this more therapeutically potent intervention, only 46% of patients could achieve an American College of Rheumatology 50% response criteria (ACR50) after 6 months of treatment, and 7% of patients experienced adverse events [8]. Therefore, finding new treatment options to achieve deep remission in RA patients, and relieve chronic fatigue and diffuse pain based on current treatment is an urgent issue for clinicians to address.

Chinese medicine is effective in the treatment of RA, with relatively mild side effects and suitable for long-term use [9]. The Wangbi granule, a well-known Chinese patent medicine, was developed by Liaoning Good Nurse Pharmaceutical (Group) Co., Ltd. (Benxi City, Liaoning Province, China) and approved by the China Food and Drug Administration (No: Liao20150121) for the treatment of RA [10]. The latest research has shown that Wangbi granule has impressive anti-inflammatory and immunomodulatory effects and can reduce pathological hyperplasia and bone destruction in damaged joints [11, 12]. As a traditional Chinese medicine preparation, Wangbi granule showed a synergistic role with methotrexate in the clinical treatment of early RA, contributing to the remission of RA [13]. However, stronger evidence is needed to confirm its further therapeutic effects in RA that has been treated but not yet reached a complete remission. This will help clinicians to better manage the course of RA patients.

In conclusion, the investigators will conduct this randomized controlled trial to evaluate the efficacy and safety of Wangbi granule in RA patients who did not achieve deep remission despite treatment with tofacitinib and methotrexate.

## Methods

### Study design

**Fig. 1:**
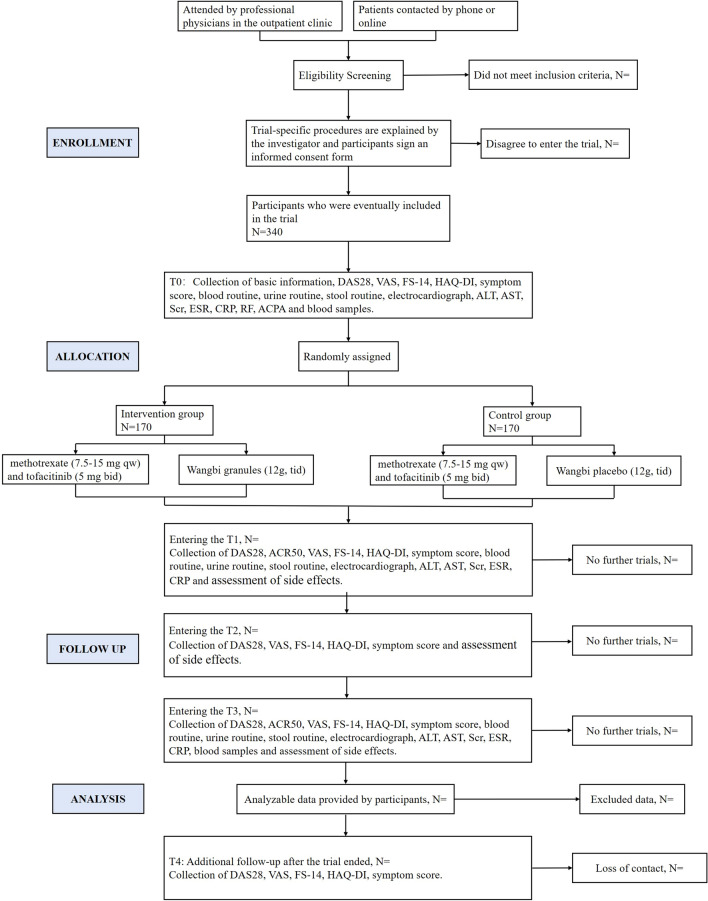
The flow diagram of the trial. T0, 0–1 day treatment (baseline); T1, 4 week treatment; T2, 8 week treatment; T3, 12 week treatment; T4, 1 month after end of treatment; *DAS28* disease activity score derivative for 28 joints, *ACR50* American College of Rheumatology 50% response criteria, *VAS* visual analogue scale for pain, *FS-14* fatigue scale-14, *HAQ-DI* health assessment questionnaire disability index, *ALT* alanine aminotransferase, *AST* aspartate aminotransferase, *Scr* serum creatinine, *ESR* erythrocyte sedimentation rate, *CRP* C-reactive protein, *RF* rheumatoid factors, *ACPA* anti-citrullinated protein antibodies

The clinical study of Wangbi granule for RA is a multicenter, randomised, triple-blind, placebo-controlled superiority trial. The study flow is designed strictly according to the CONSORT Statement and SPIRIT Statement [14, 15] (Fig. 1). The study will restrict the enrollment of participants, use strict randomization and blinding, and apply some statistical methods to prevent possible bias in these ways.

### Eligibility criteria

Patients aged 18–75 years who meet the 1987 or 2010 international diagnostic criteria for RA are eligible for the study [16, 17]. Patients need to meet the diagnostic criteria (2018 edition) for “kidney deficiency and dampness” and “stasis blocking channels” in Chinese medicine [18]. The disease activity score derivative for 28 joints (DAS28) [19] score should be 2.6 to 3.2. The use of methotrexate (7.5–15 mg, quaque week, dose changes with disease) in combination with tofacitinib (5 mg, bis in die) for more than 12 weeks prior to recruitment is required. Before recruitment, clear notification and signing of informed consent are required.

Exclusion criteria include: taking immunosuppressants other than methotrexate and tofacitinib in the 3 months prior to recruitment; combination of other severe multi-system damaging diseases; presence of cognitive impairment, psychosomatic disorders, peripheral neurological disorders; those who are pregnant, breastfeeding, or have a planned pregnancy; overweight, allergic to the study drug, or enrolled in another clinical trial; other conditions in which the investigator believes the patient should not participate in the trial. The complete list of inclusion and exclusion criteria is provided in Table 1.

**Table 1:** Inclusion and exclusion criteria of the trial

Inclusion criteria	Exclusion criteria
► Age 18–75 years ► Meeting the 1987 ACR or 2010 ACR/EULAR diagnostic criteria ► Meeting the diagnostic criteria for “kidney deficiency and dampness” and “stasis blocking channels” in Chinese medicine ► DAS28 score of 2.6 to 3.2 ► Regularly taking methotrexate (7.5–15 mg qw) and tofacitinib (5 mg bid) before enrollment and stable treatment regimen for more than 12 weeks ► Voluntary participation and signed written informed consent	► Patients taking immunosuppressive drugs other than methotrexate and tofacitinib within 3 months prior to enrollment ► Organ transplant recipients, patients with malignant tumors, patients with heart, brain, liver (ALT/AST > 3 times the normal upper limit), kidney (Ccr < 60 ml/min) and other important organ function impairment or hematological system diseases ► Psychiatric disorders such as cognitive disorders, depression, anxiety disorders, somatic dysfunction, cerebral infarction, cerebral hemorrhage, epilepsy, TIA, myelitis, demyelinating lesions and other central neurological disorders, or peripheral neurological disorders such as restless legs syndrome. peripheral neurological disorders such as restless legs syndrome ► Women and men who are pregnant or breastfeeding or who are planning a pregnancy within the next 6 months; during the trial or within 1 month of the last dose Women of childbearing age who are unable or unwilling to use adequate contraception, or whose spouse is unwilling to use contraception, within 1 month of the last dose or within 1 month of the last dose are unwilling to use contraception ► Persons with a BMI greater than 35 (kg/m^2^), allergic to the test drug, or participating in other clinical trials ► Other conditions deemed by the investigator to be inappropriate for trial participation (out-of-town patients unable to be followed up, etc.)

*ACR* American college of rheumatology, *EULAR* European league against rheumatism, *DAS28* disease activity score derivative for 28 joints, *qw* quaque week, *bid* bis in die, *DMARDs* disease-modifying antirheumatic drug, *ALT* alanine aminotransferase, *AST* aspartate aminotransferase, *Ccr* creatinine clearance rate, *TIA* transient ischemic attacks, *BMI* body mass index

Participants may decide to withdraw from the trial at any time for any reason of their own choosing. The investigators may also ask the participant to withdraw from the trial for rational reasons. All participants who have completed the informed consent form and are screened and qualified to enter the randomized trial, regardless of when and why they withdrew, will be treated as dislodged cases as long as they do not complete the observation period specified in the protocol.

### Study setting

This trial is led by the Traditional Chinese Medicine Department of Rheumatology at China-Japan Friendship Hospital and will be conducted at 6 hospitals in China (China-Japan Friendship Hospital, Peking Union Medical College Hospital, The First Affiliated Hospital of Anhui University of Traditional Chinese Medicine, The 980th Hospital of PLA Joint Logistics Support Force, The Second Affiliated Hospital of Zhejiang Chinese Medical University, and The First Hospital Affiliated to Army Medical University).

### Recruitment

A total of 340 patients will be recruited from the 6 centers participating in this study. An investigator, assisted by a trained study coordinator at each participating center, will identify potential eligible patients based on the eligibility criteria. They will work with the attending physician to determine whether the patient is eligible for the trial. Patients who travel to outpatient or inpatient settings for RA are evaluated for eligibility by an investigator who collects relevant information face-to-face with the patient’s verbal consent at the time of treatment. If RA patients are recruited from posters, the internet, etc., eligibility will be assessed over the phone. After the patient is deemed to meet the criteria for participation, the investigator will inform the study protocol in detail, at which point potential participants can ask their questions and requests, and after formally signing written informed consent, they are enrolled in the trial as participants.

### Interventions

After being recruited, patients will be randomly allocated to either the intervention group or the control group (in a 1:1 ratio).

Based on the treatment of methotrexate (7.5–15 mg, quaque week, dose changes with disease) (Shanghai Sine Pharmaceutical, Shanghai, China) and tofacitinib (5 mg, bis in die) (Qilu Pharmaceutical, Shandong, China), patients in the intervention group will be given Wangbi granule (12 g, ter in die) (Good Nurse Pharmaceutical, Liaoning, China) and patients in the control group will be given Wangbi placebo (12 g, ter in die) (Good Nurse Pharmaceutical, Liaoning, China). To reduce the adverse effects of methotrexate, folic acid (10 mg, quaque week) (Lisheng Pharmaceutical, Tianjin, China) will be used in combination with all treatments.

All the botanical or zoological drugs that make up the Wangbi granule are explained in Table 2. All trial drugs will be manufactured and supplied by Liaoning Good Nurse Pharmaceutical (Group) Co., Ltd. (Benxi City, Liaoning Province, China) and tested by its quality management department to meet the Pharmacopoeia of China (2020 edition) grade standards. Voucher samples of all drugs will be stored at China-Japan Friendship Hospital (Beijing, China). The drug preparation and packaging will be operated by the pharmacy of Good Nurse Pharmaceutical in a fully enclosed production line. All ingredients will be mixed in the original dosage, steeped in pure water for 0.5 h, heated to boiling, decocted for 2 h, filtered from the aqueous extract, then the residue will be added to pure water and decocted for 1 h. The two liquid extracts will be combined, settled, filtered and concentrated into a thick paste (relative density 1.32–1.35, 50 °C), with a certain amount of soluble starch and dextrin as auxiliary materials to assist in forming, and processed into 1000 g concentrated granules by spray drying. The final Wangbi granule is equivalent to 27.88 g of the original drugs per 12 g (ter in die, a total of about 83.63 g per day) and sealed with aluminum-plastic composite film (BOPP/AL/CPE) packaging material. The Wangbi granule needs to be dissolved in hot water for each use. The placebo consists of one tenth of the dosage of Wangbi granule, starch, caramel color and bitter agent. Its appearance and odor are the same as those of Wangbi granule. The study drugs are from the same batch, purchased from Good Nurse Pharmaceutical (Liaoning, China), coded and kept under lock and key by the drug administrator in a special cabinet at room temperature and dry place. On the day of enrollment, patients will be given these medications.

**Table 2:** Components of Wangbi granule (intervention drug)

Chinese name	Chinese Pinyin	Scientific name	Family	Part used	Original dosage (g)	Daily dosage(g)
生地黄	Sheng Di Huang	*Rehmannia glutinosa* (Gaertn.) DC.	Orobanchaceae	Dried root	196	7.056
熟地黄	Shu Di Huang	*Rehmannia glutinosa* (Gaertn.) DC.	Orobanchaceae	Steamed root	196	7.056
续断	Xu Duan	*Dipsacus asper* Wall. ex DC.	Caprifoliaceae	Dried root	147	5.292
附子	Fu Zi	*Aconitum carmichaeli* Debeaux	Ranunculaceae	Dried lateral root tuber	147	5.292
独活	Du Huo	*Angelica biserrata* (R.H.Shan & C.Q.Yuan) C.Q.Yuan & R.H.Shan	Apiaceae	Dried root	98	3.528
骨碎补	Gu Suibu	*Drynaria roosii* Nakaike	Polypodiaceae	Dried rhizome	147	5.292
桂枝	Gui Zhi	*Neolitsea cassia* (L.) Kosterm.	Lauraceae	Dried young branch	98	3.528
淫羊藿	Yin Yanghuo	*Epimedium sagittatum* (Siebold & Zucc.) Maxim.	Berberidaceae	Dried aerial parts	147	5.292
防风	Fang Feng	*Saposhnikovia divaricata* (Turcz. ex Ledeb.) Schischk.	Apiaceae	Dried root	98	3.528
威灵仙	Wei Lingxian	*Clematis chinensis* Osbeck	Ranunculaceae	Dried root and rhizome	147	5.292
白芍	Bai Shao	*Paeonia lactiflora* Pall.	Paeoniaceae	Dried root	117.67	4.236
狗脊	Gou Ji	*Cibotium barometz* (L.) J.Sm.	Cyatheaceae	Dried rhizome	147	5.292
皂角刺	Zao Jiao Ci	*Gleditsia sinensis* Lam.	Fabaceae	Dried spine	98	3.528
知母	Zhi Mu	*Anemarrhena asphodeloides* Bunge	Asparagaceae	Dried rhizome	147	5.292
伸筋草	Shen Jincao	*Lycopodium japonicum* Thunb.	Lycopodiaceae	Dried herb	98	3.528
红花	Hong Hua	*Carthamus tinctorius* L.	Asteraceae	Dried flower	98	3.528
羊骨	Yang Gu	*Capra hircus* L.	Caprinae	Dried bone	196.44	7.072

Except for the experimental drugs, the participants are not allowed to use other botanical, zoological and Western medicines with immunosuppressive or immunomodulatory effects. All treatments that may improve RA, such as acupuncture and massage, will be banned during the trial. If a patient has special circumstances during the observation period, for instance. In that case, when pain is unbearable, drugs such as low-dose NSAIDs can be used in combination under the direction of the investigator, and all drug use outside the study protocol must be recorded in detail.

The participants will take the drugs daily for 12 consecutive weeks under self-monitoring. During the course of the medication intervention, we will strictly ensure that participants are adequately adherent. After the participant has formally decided to participate in the clinical study, the investigator will explain the entire trial process, including the time points at which various types of information will be collected, the meaning of every terminology in the questionnaire, the frequency and method of how participants need to take drugs. Participants are required to fill out daily health monitoring forms to record medication intake and changes in the condition so that the investigator can check. Participants will be asked to bring all leftover drugs to every follow-up visit to check their compliance. Investigator faithfully recorded the number of drugs received, taken and returned by the participant and promptly recorded them on the case report form. Once a participant enrolled in the study, the drugs used and the tests involved will be covered by the study center and the participant will be reimbursed a certain amount of grants when the trial is completed in full. Through these incentives, participants will maximize their enthusiasm for the trial, which is expected to improve in RA clinical conditions.

### Outcomes

The efficacy of Wangbi granule will be observed and the main outcome is DAS28 [19], assessed by a dedicated rheumatologist (associate senior physician or better). The changes in the DAS28 score will be recorded during the observation period. Moreover, participants are considered to have achieved deep clinical remission when the calculated DAS28 score is lower than 2.6.

Secondary outcomes include patient-reported outcomes (symptom score) [18], ACR50 [20], fatigue scale-14 (FS-14) [21], visual analogue scale for pain (VAS) [22], health assessment questionnaire disability index (HAQ-DI) [23] and biological markers [24] such as CRP and ESR. Specific secondary outcomes are shown in Table 3.

**Table 3:** Secondary outcomes of the trial

Secondary outcomes	
ACR50	ACR response was used to evaluate improvement in RA. ACR response requires the collection of the degree of improvement in the number of joints with pressure pain and the degree of improvement in the number of joints with swelling and the degree of improvement in 3 of the following 5 items: patient’s assessment of pain, patient’s overall assessment of disease activity, physician’s overall assessment of disease activity, patient’s assessment of physical function (HAQ), and values of acute phase reactants (ESR, CRP). Percentage improvement in each indicator = (pre-treatment value − post-treatment value)/pre-treatment value * 100% ACR20: ≥ 20% improvement in joint pain and swelling, and ≥ 20% improvement in at least 3 of the other 5 items ACR50: ≥ 50% improvement in joint pain and swelling, and ≥ 50% improvement in at least 3 of the other 5 items ACR70: ≥ 70% improvement in joint pain and swelling, and ≥ 70% improvement in at least 3 of the other 5 items
VAS	Patients’ joint pain levels were evaluated using the VAS, expressed on a ten-point scale, with higher scores indicating more intense pain Face-to-face or telephone follow-ups were conducted at baseline, 4 weeks, 8 weeks, and 12 weeks of treatment, and patients were scored based on their subjective perceptions
FS-14	FS-14 is used to measure the patient’s fatigue level. FS-14 was divided into 2 dimensions: physical fatigue (8 items) and mental fatigue (6 items). The scoring method is based on a 2-point scale, with 0 points for “No” and 1 point for “Yes”, and reverse scoring for items 10, 13 and 14, with a total score of 0 to 14 points, with higher scores representing heavier fatigue Face-to-face or telephone follow-ups were conducted at baseline, 4 weeks, 8 weeks, and 12 weeks of treatment, and patients were scored based on their subjective perceptions
HAQ-DI	The HAQ-DI was used to assess the overall functional status of the patients. The questionnaire consists of 20 questions containing 8 areas of daily activity, with higher scores on each question representing poorer functioning Face-to-face or telephone follow-ups were conducted at baseline, 4 weeks, 8 weeks, and 12 weeks of treatment, and patients were scored based on their subjective perceptions
Symptom score	The patient’s symptoms associated with RA were recorded, including joint swelling, pressure pain, activity level, diet, etc. The greater the degree of performance of each symptom, the higher the score Face-to-face or telephone follow-ups were conducted at baseline, 4 weeks, 8 weeks, and 12 weeks of treatment, and patients were scored based on their subjective perceptions
Biological markers	All patients will have their venous blood and other required specimens collected on an empty stomach in the early morning of the specified date and tested in the testing department of each research center, and the biological samples will be frozen in a special refrigerator in China-Japan Friendship Hospital Routine blood, urine, and stool tests were performed at baseline, 4 weeks, and 12 weeks of the trial Electrocardiograph, ALT, AST, and serum creatinine were tested at baseline, 4 weeks, and 12 weeks of the trial to monitor the safety of drug administration ESR and CRP were measured at baseline and 12 weeks of the trial to monitor inflammation levels RF and ACPA were tested during the baseline period of the trial to determine the patient’s antibody levels Additional blood samples were collected at baseline, week 12 of the trial and used to test for various proteins and genes such as inflammatory cytokines

*ACR50* American College of Rheumatology 50% response criteria, *VAS* visual analogue scale for pain, *FS-14* fatigue scale-14, *HAQ-DI* health assessment questionnaire disability index, *ALT* alanine aminotransferase, *AST* aspartate aminotransferase, *ESR* erythrocyte sedimentation rate, *CRP* C-reactive protein, *RF* rheumatoid factors, *ACPA* anti-citrullinated protein antibodies

### Safety

Medications including methotrexate, tofacitinib, and Wangbi granule are all commonly used for RA and are not expected to cause unacceptable side effects compared to their therapeutic effects. If an adverse reaction is suspected, treatment will be stopped immediately with the permission of the Principal Investigator. During the trial period, investigator will provide participants with free trial drugs. Participants will have access to some free tests and receive a set amount of subsidy.

An adverse event is defined as any adverse medical event that occurs between the time the participant signs the informed consent and the last follow-up visit, whether or not it is causally related to the study drug. Adverse events will be monitored continuously during the treatment period, regardless of whether adverse events are related to the trial drug, the investigators should fill out the record form truthfully and in detail. A serious adverse event, such as a new or previous exacerbation of the patient’s disease during treatment, must be reported within 24 h. Termination of the trial should be considered when additional immunosuppressive drugs or other medications, surgery or other physical therapy associated with the improvement of RA are indeed required. Such a sample will be treated as an off-case. The study team will continue to track the progress of adverse events.

### Allocation and blinding

A central randomization system will be applied in this study, with an independent contract research organization (CRO) implementing the randomized assignment and drug dispensing in each subcenter. Participants will be randomized to either the intervention or control group, and stratified block group randomization will be used for participant randomization. In the random assignment process, participants are first allowed to enter the zone group and then are randomly allocated. The length of the zone group is set to 4, and the six permutations obtained are designated as AABB, ABAB, ABBA, BAAB, BABA, BBAA, with A and B representing the intervention or control group, whose exact meaning is known only to the designer. Subsequently, a string of random numbers is generated by SPSS software, and the allocation sequence is obtained by selecting numbers between 1 and 6 and arranging the district groups in the order of random numbers. Once each subcenter has determined the eligibility of participants and obtained the written consent to participate in the trial, a number will be requested from the randomization manager. The complete sequence of generated assignments is kept at the CRO, and the designer will place them in sequentially numbered sealed opaque envelopes and store them in a locked cabinet.

Researchers, participants and statistical analysts will be blinded to group assignment. After all cases are included, all clinical data will be entered into the database and verified, and the data will be locked after confirmation of accuracy. Groups A and B will be divided into two groups at the first unblinding, but it is not clear which group is the intervention group. After the statistical analysis, the results will be processed and the second unblinding will be carried out to find out whether group A and group B represent the intervention group or the control group. If a serious adverse event or unexpected adverse reaction occurs during the trial, the patient’s blinding status will be broken with the consent of the Principal Investigator (PI), and all such cases will be reported to the Clinical Research Ethics Committee of the China-Japan Friendship Hospital.

### Participant timeline

Participants in the trial are required to be recorded four times, with visits at enrollment (in the hospital), 4 weeks (in the hospital), 8 weeks (by telephone follow-up), and 12 weeks (in the hospital) after intervention, each taking approximately 15–20 min. In addition, participants are asked to have an additional telephone follow-up visit 1 month after completion of the study, regardless of the type of intervention (Table 4).

**Table 4:** Time schedule of enrolment, intervention and outcome measures of the trial

Timepoint	Study period					
	Screeningstage	T0: 0–1 day Treatment (Baseline)	T1: 4 week treatment	T2: 8 week treatment	T3: 12 week treatment	T4: 1 month after end of treatment
Face-to-face meeting		**√**	**√**		**√**	
Enrolment						
Eligibility screening	**√**					
Informed consent	**√**					
Allocation	**√**					
Intervention						
Wangbi granules/Placebo		**√**	**√**	**√**	**√**	
Outcomes and measures						
Basic information		**√**				
DAS28		**√**	**√**	**√**	**√**	**√**
ACR50			**√**		**√**	
VAS		**√**	**√**	**√**	**√**	**√**
FS-14		**√**	**√**	**√**	**√**	**√**
HAQ-DI		**√**	**√**	**√**	**√**	**√**
Symptom score		**√**	**√**	**√**	**√**	**√**
Blood routine		**√**	**√**		**√**	
Urine routine		**√**	**√**		**√**	
Stool routine		**√**	**√**		**√**	
Electrocardiograph		**√**	**√**		**√**	
ALT/AST/Scr		**√**	**√**		**√**	
ESR/CRP		**√**	**√**		**√**	
RF/ACPA		**√**				
Blood samples		**√**			**√**	
Assessment of side effects			**√**	**√**	**√**	

*DAS28* disease activity score derivative for 28 joints, *ACR50* American College of Rheumatology 50% response criteria, *VAS* visual analogue scale for pain, *FS-14* fatigue scale-14, *HAQ-DI* health assessment questionnaire disability index, *ALT* alanine aminotransferase, *AST* aspartate aminotransferase, *Scr* serum creatinine, *ESR* erythrocyte sedimentation rate, *CRP* C-reactive protein, *RF* rheumatoid factors, *ACPA* anti-citrullinated protein antibodies

### Sample size calculation

The study used superiority design approach. Due to the lack of clinical studies of methotrexate and tofacitinib in combination with Wangbi granule, we refer to the previous studies of methotrexate in combination with Wangbi granule for the treatment of RA [25]. In order to observe the deep remission effect of the Wangbi granule on RA condition, the final DAS28 score of the intervention group needs to be at least 0.5 points lower than that of the control group, so the cut-off value δ takes − 0.5. We adopted single-sided calculation whereα is 0.025, β is 0.2, and the sample size is 1:1, then the single group sample size of 155 cases is obtained. Considering a shedding rate of 10%, 170 cases are needed in each group, and the final total sample size is 340.According to the plan, as the lead center, China-Japan Friendship Hospital will complete the intake of 90 samples, and the remaining 5 centers will equally distribute the remaining 250 samples.

### Statistical analysis

All analysis and data visualization will be performed using IBM SPSS (Version 25.0), R (Version 4.1.1).

Baseline data will be displayed using descriptive statistics (eg, means, SD, median, IQR, proportions). We will test the group balance achieved by randomization through appropriate statistical tests (eg, Student’s t tests, Pearson’s χ^2^ tests). Outcomes with a continuous distribution will be compared between groups using the mean (Student’s t test) and Pearson’s χ^2^ test will be used for binary categorical data where categorical variables are expressed as frequencies and percentages and continuous variables are expressed as means and SD.

Preliminary statistical analysis of the primary outcome data will be performed according to the intention-to-treat principle, and the changes in DAS28 will be compared between patients treated with Wangbi granule and Wangbi placebo, and report the rate of deep remission. Further, two-way repeated measures analysis of variance (ANOVA) will be used to examine any interaction effects of the outcome variables between the two groups at the four time points. Mauchly’s sphericity test will be used to examine the spherical assumptions of the repeated measures ANOVA. Bonferroni-corrected post hoc multiple comparisons will be used to examine simple effects between groups at different time points and at different time points for each group.

Sensitivity analysis will be an essential part of the statistical analysis process to complement the interpretation of the results. Depending on the specifics of the data, the full samples will be divided into subgroups based on information about additional drugs (e.g. NSAIDs), co-existing underlying conditions (e.g. hypertension, diabetes), long-term use of other drugs, etc. The calculations will be re-run in each subgroup to verify the validity of the results. When necessary, a per protocol set (PPS) analysis will be part of the sensitivity analysis. If any variable has no more than 20% missing values, the MICE package in R will be used for multiple interpolation to replace it. For the statistical process, *P* < 0.05 is considered statistically significant.

### Data management

Investigators and participants obtain the required information in person or by telephone, and investigators will consult the hospital’s medical records system to complete additional details. All the data obtained are recorded in the special paper case report form. The information obtained from the last recording will be used as the final data for participants who withdrew. After the CRO collects the paper documents returned by each research center, these data are stored in a special database for unified management. Epidata software will be used to establish the corresponding database, and the data will be entered synchronously by different data managers, using the two-entry method. The CRO will be in charge of data management. The biological sample are stored in the bio-sample bank of China-Japan Friendship Hospital.

### Quality control

Due to the need to collect data on some of the more subjective scales, such as symptom score, FS-14, etc. The PI will provide adequate training to the investigators at each sub-center before the start of the trial to ensure that the investigators understand the complete study protocol and operational methods. The principal investigators of each research center must be an associate senior physician or better. Two investigators will complete the participant’s rating at each follow-up visit at the same time, and the average will be taken as the final record to reduce the bias of subjective assessment. A third investigator will assist in the assessment when the difference in results is too wide. After participants enrolled in the trial, the investigators will educate them to ensure that participants know exactly how to judge their condition and score subjectively.

Supervisors are appointed by the PI to ensure that the rights and interests of subjects in clinical trials are protected, that data in trial records and reports are accurate and complete, to oversee the implementation of clinical trial protocols, drug clinical trial management norms and relevant regulations, and to conduct regular on-site supervision visits to each center. For objective indicators, paper reports need to be retained for subsequent verification.

The trial is monitored by the Clinical Research Ethics Committee of the China-Japan Friendship Hospital. Moreover, the third-party independent CRO will perform quality control throughout the trial process.

## Discussion

We expect that the current study can address a major concern in the field of RA treatment. Most patients still suffer from persistent symptoms and elevated inflammatory factors after a stronger medication regimen, in which case simply increasing the drug dose or continuing the combination with other DMARDs does not continue to improve the condition, but rather raises the risk of side effects [26]. When Wangbi granule is newly added to the management of RA as a primary therapeutic intervention, there may be additional benefits for patients. This will further guide us to develop improved treatment and home management guidelines to better respond to changes in patient health status.

In addition to assessing clinical efficacy and providing evidence-based medical evidence, this study also attempts to provide assistance in the precise treatment of RA through the detection of biological samples (e.g., inflammatory factors in blood). In the course of systemic anti-inflammatory treatment, synovitis-targeted therapies provide support for improvement, and most of the biologically targeted agents that exist today target key cytokines [27], mainly TNF, IL-6 family, etc. Chinese medicine may play a more important role at the pathophysiological level because of its unique multi-targeted effects, and studies have proved that Wangbi granule can modulate immune and cytokines and intervene at the proteomic level [28]. This trial can further explore the possible molecular regulatory network on this basis, provide therapeutic targets from a multi-omics perspective, and provide practical basis for precise treatment of integrated traditional Chinese and Western medicine.

Inevitably, however, there are some limitations in this study. In the judgment of the disease, most of the scores are based on the subjective feelings of the patients, which may have some bias. In addition, longer-term follow-up will not be designed (participants in this study will complete 3 months of treatment and 1 month of follow-up after treatment) due to cost constraints.

## Conclusion

This study is a multicenter, triple-blind, randomized, placebo-controlled trial that potentially provides an effective treatment strategy for the integration of traditional Chinese and Western medicine in the treatment of RA.

## Data Availability

The original contributions presented in the study are included in the article, further inquiries can be directed to the corresponding authors.

## Abbreviations

ACR50: American College of Rheumatology 50% response criteria
bDMARDs: Biologic disease-modifying antirheumatic drugs
CRO: Contract research organization
CRP: C-reactive protein
csDMARDs: Conventional synthetic disease-modifying antirheumatic drugs
DAS28: Disease activity score derivative for 28 joints
ESR: Erythrocyte sedimentation rate
FS-14: Fatigue scale-14
HAQ-DI: Health assessment questionnaire disability index
NSAIDs: Non-steroidal antiinflammatory drugs
PI: Principal Investigator
PPS: Per protocol set
RA: Rheumatoid arthritis
VAS: Visual analogue scale for pain
